# Building Expertise Across Borders: The IAEA’s Expanding Digital Education in Nuclear Medicine and Radiology

**DOI:** 10.3390/diagnostics16121837

**Published:** 2026-06-13

**Authors:** Amir Eskander, Francesco Giammarile, Arthur Colaco Pires de Andrade, Anita Brink, Roberto C. Delgado Bolton, Enrique Estrada Lobato, Peter Knoll, Miriam Mikhail-Lette, Kgomotso Mokoala, Oscar Rollgeiser, Diana Paez

**Affiliations:** 1 Nuclear Medicine and Diagnostic Imaging Section, Division of Human Health, Department of Nuclear Sciences and Applications, International Atomic Energy Agency, 1400 Vienna, Austria; amir.m.eskander@med.asu.edu.eg (A.E.); a.andrade@iaea.org (A.C.P.d.A.); a.brink@iaea.org (A.B.); e.estrada-lobato@iaea.org (E.E.L.); m.mikhail-lette@iaea.org (M.M.-L.); k.mokoala@iaea.org (K.M.); oscar@rollgeiser.de (O.R.); d.paez@iaea.org (D.P.); 2 Department of Diagnostic Radiology, Interventional Radiology and Molecular Imaging, Faculty of Medicine, Ain Shams University, Cairo 1181, Egypt; 3 Department of Diagnostic Imaging (Radiology) and Nuclear Medicine, University Hospital San Pedro and Centre for Biomedical Research of La Rioja (CIBIR), 26006 Logroño, Spain; rbiolton@gmail.com; 4 Servicio Cántabro de Salud, 39011 Santander, Spain; 5 Dosimetry and Medical Radiation Physics Section, Division of Human Health, Department of Nuclear Sciences and Applications, International Atomic Energy Agency, 1400 Vienna, Austria; p.knoll@iaea.org

**Keywords:** digital education, nuclear medicine, radiology, e-learning, continuing professional development, capacity building, International Atomic Energy Agency, diagnostic imaging

## Abstract

Diagnostic imaging is central to clinical decision-making across many care pathways, yet the expertise needed to use these images well is unevenly distributed across health systems, with workforce limitations identified as a major barrier to equitable access, particularly in low- and middle-income countries. Digital education has emerged as one response to this gap, offering scalability, asynchronous and just-in-time access, and the cost-efficiency required for global deployment. This paper examines the digital education portfolio of the International Atomic Energy Agency’s Nuclear Medicine and Diagnostic Imaging Section, hosted mainly on the open-access Human Health Campus, which in 2025 recorded approximately 45,800 active users and 150,000 views across 159 countries. The portfolio combines structured e-learning courses, interactive webinars, virtual conference access through the Livestream programme, and a broader repository of publications, teaching cases, and reference resources, supported by an internal e-learning framework and learning management system infrastructure. Partnerships with international scientific societies further extend the reach of expert knowledge and professional exchange. The paper argues that these initiatives are best understood not as content delivery alone but as a coordinated strategy to support diagnostic quality at the level of the practising physician, extending access to expertise and strengthening the conditions for better practice, while remaining a complement to, rather than a substitute for, supervised clinical training.

## 1. Introduction

Diagnostic imaging and nuclear medicine sit at the centre of clinical decision-making across many care pathways. Yet the expertise needed to use these diagnostic tools is unevenly distributed globally, with direct consequences for whether patients receive the right examination at the right time for the right clinical question, and whether the imaging findings translate into appropriate management.

Equitable access to diagnostic imaging and nuclear medicine is increasingly recognised as foundational to effective health care systems. In 2025, the Seventy-eighth World Health Assembly adopted resolution WHA78.13 on strengthening medical imaging capacity, recognising imaging as vital for the diagnosis and treatment of both communicable and noncommunicable diseases, and calling for improved equitable access to safe, effective, and affordable imaging services globally as well as urging Member States to commit to adequate education and continuous training of imaging professionals [1]. Nevertheless, sustainable expansion remains insufficient: alongside infrastructure gaps and equipment shortages, limitations in workforce capacity represent a major constraint. The Lancet Oncology Commission on medical imaging and nuclear medicine identifies “insufficient human resources” and “inadequate and insufficient programmes for training personnel” as barriers restricting access to imaging and nuclear medicine for cancer, with substantial disparities across low-income and middle-income countries (LMICs) [2]. This gap in training is further compounded by the increasing complexity and rapid evolution of the field, this includes hybrid imaging, theranostics, and artificial intelligence (AI) applications, requiring both initial training and ongoing professional development.

Historically, professional training in radiology and nuclear medicine has been grounded primarily in in-person, institution-based models, including residency programmes and fellowships. While these approaches remain indispensable for hands-on clinical training, they are limited in scale, cost, and geographic reach [3]. In contrast, digital learning technologies can support engagement across borders at scale, enabling learners to participate in asynchronous activities independent of time and location, as well as draw on focused resources in a just-in-time manner aligned with immediate clinical needs. Digital approaches also offer favourable cost-efficiency, as high-quality resources can be developed once and deployed globally through digital platforms with minimal recurrent cost. Reflecting this rationale, the Lancet Oncology Commission recommends investment in digital solutions and virtual platforms to enable rapid scale-up of training in LMICs as part of broader efforts to expand imaging capacity [2]. The educational evidence base supports cautious optimism about this approach: a meta-analysis of internet-based learning has shown positive effects compared with no intervention, with more variable effects relative to traditional formats [4], and a synthesis of systematic reviews on continuing medical education (CME) has concluded that well-designed activities can improve physician performance, particularly when they are interactive, draw on multiple educational methods, and involve repeated exposure [5]. These findings support the use of digital education for continuing professional development, while cautioning against overclaiming its direct effects on patient outcomes or diagnostic error reduction.

Within this context, the International Atomic Energy Agency (IAEA), whose mandate includes supporting the peaceful application of nuclear technologies for health [6], delivers health-related capacity-building activities through its Division of Human Health [7]. The Nuclear Medicine and Diagnostic Imaging Section [8] focuses specifically on strengthening nuclear medicine and radiology services—both diagnostic and therapeutic—across Member States, working through technical cooperation projects, coordinated research, training, quality management, and guidance documents. Digital education is part of the Section’s broader capacity-building mandate: it is one of the ways it helps establish, improve, and sustain services, rather than a stand-alone technology-mediated initiative. Recognising the expanding role of digital education in addressing workforce and access limitations, the Section has progressively invested in developing digital educational tools and resources, either through the Human Health Campus (HHC)—the Division’s own digital platform—or through complementary initiatives delivered in collaboration with professional societies and other organisations.

This paper examines the structure, scope, and evolution of the Section’s digital education initiatives, and argues that they are best understood not as content delivery alone but as a coordinated strategy to support diagnostic quality at the level of the practising physician, strengthening the conditions under which nuclear medicine physicians and radiologists choose, perform, interpret, and communicate imaging across diverse health systems. The paper also discusses key strategic and operational challenges within the contemporary global landscape, highlighting the role of an international organisation in supporting workforce development and equitable access to educational resources across Member States.

## 2. Open-Access Infrastructure for Radiation Medicine Education: The Human Health Campus

The Human Health Campus (HHC; https://www.iaea.org/resources/hhc; accessed on 26 March 2026) serves as the principal structured platform hosting the Division of Human Health’s digital education resources and initiatives. Originally launched in 2010, the HHC was designed as a centralised virtual campus to support professionals across radiation medicine—encompassing nuclear medicine, diagnostic imaging, radiation oncology, and medical radiation physics—as well as nutrition [9]. Its establishment marked a shift from previously isolated digital materials distributed through multiple and fragmented channels toward an integrated approach that aggregates diverse educational formats and digital learning initiatives within a unified platform. Since its initial description in 2013 [9], the HHC has expanded substantially: the structured e-learning portfolio has grown from a single course to thirty, the user interface and authoring tools have been modernised in line with current web standards, and integration with the IAEA’s learning management system now supports enrolment, certification, and structured data collection.

The HHC operates under a free and open-access model. This approach is consistent with the IAEA’s mandate as well as with global policy calls aimed at reducing disparities in access to specialist training globally and locally. It is particularly significant because evidence indicates that open-access medical education without financial barriers can facilitate wider participation, expand opportunities for continuing professional development, and support a more equitable distribution of knowledge resources [10,11]. This is especially relevant in LMICs and resource-constrained settings, where subscription-based or paywalled educational material may be financially prohibitive.

The open-access structure of the HHC therefore represents a deliberate strategic choice by the IAEA to promote more equitable access to specialist knowledge across Member States. This positioning distinguishes the HHC from the major society-led educational ecosystems in nuclear medicine and radiology—for instance the European Association of Nuclear Medicine (EANM), the Radiological Society of North America (RSNA), and the European Society of Radiology (ESR)—which typically operate on membership or subscription models with selected free content and whose offerings, while internationally engaged, are shaped primarily by the clinical and regulatory context of the region they are based in. The HHC complements these ecosystems by providing a fully open-access, globally oriented platform specifically designed to support capacity-building across IAEA Member States, with a particular focus on LMICs where access to society-based education may be limited by cost, membership eligibility, or regionally oriented content.

Platform utilisation metrics (acquired using Google Analytics 4) further illustrate the global reach of the HHC. In 2025, the platform recorded approximately 45,800 active users (unique users with at least one engaged session or first visit) and 150,000 total views, with repeat visits by the same user counted as additional views rather than as additional active users. Over the six-year period analysed, visits originated from 159 countries, of which 105 (66%) were classified as LMICs and 54 (34%) as high-income countries (according to the World Bank country and lending groups [12]); in terms of number of visits, approximately 55% of total visits originated from LMIC settings and 45% from high-income countries. Although these figures do not constitute outcome measures, they indicate that the HHC is a globally accessed digital resource across geographically and economically diverse settings.

Within the Nuclear medicine and radiology section of the HHC, a broad range of digital learning resources is hosted, including structured e-learning courses, interactive modules, webinars, recorded lectures, downloadable slide-based lecture materials, and teaching cases. Each resource type can be browsed either as a consolidated list or by specialty areas within nuclear medicine and radiology, supporting both topic-based and format-based exploration. Table 1 summarises these resource categories, their scale on the HHC, the primary educational purpose of each, and representative examples of how they support nuclear medicine physicians and radiologists in their clinical practice.

## 3. Structured E-Learning: Building Physician Reasoning at Scale

While the HHC hosts a diverse range of educational resources, structured e-learning courses represent a central pillar of the Section’s digital education strategy, providing more comprehensive and sequential coverage of major topics. Unlike shorter, standalone materials, they are designed to address broader clinical and technical concepts in a systematic manner, enabling learners to engage with complex topics through organised, curriculum-oriented progression.

### 3.1. Strategic Development of Structured E-Learning Courses

The e-learning portfolio has expanded substantially, from a single course launched in 2011 to thirty courses today, covering diverse areas within nuclear medicine and radiology. This growth reflects a deliberate priority within the Section’s digital learning strategy, leveraging the format’s benefits for both learners and the institution.

For learners, they can engage with content at their own pace, independent of time and location, accommodating their different clinical schedules with the added benefit of just-in-time learning support, enabling them to consult focused thematic material in response to immediate clinical needs. In addition, their interactive nature enhances engagement compared with more passive educational formats such as books or recorded videos [24]. Embedded questions and knowledge checks can promote more active cognitive processing, while structured navigation pathways can facilitate efficient content navigation, together improving usability and learner experience.

From an institutional perspective, structured e-learning offers two important strategic advantages: scalability and agility. Once developed, a course can be deployed globally and simultaneously across Member States irrespective of geography or time zone, allowing efficient dissemination of standardised, high-quality educational content while minimising recurrent delivery costs [2]. Furthermore, the intermediate scope of structured e-learning courses makes them particularly suitable for targeted addressing of defined knowledge gaps or emerging clinical topics without requiring the development of full curricular programmes. This is particularly valuable in rapidly evolving fields such as nuclear medicine and diagnostic imaging, where new technologies or clinical applications may require timely educational responses.

The format also demonstrates adaptability in content design. The 2025 e-learning module PET-CT for the Management of Cancer Patients: a Review of the Existing Evidence [12] is an adaptation of IAEA Human Health Series No. 45 of the same title [25], providing learners with guidance on appropriate PET/CT use in different oncological scenarios in a visual, easy-to-navigate format, with the addition of integrated case examples drawn from the HHC PET/CT gallery. This transformation enhances usability, facilitates structured navigation of evidence-based recommendations, and promotes more active engagement with the material.

In addition to stand-alone delivery, the developed e-learning modules have also supported blended learning models. Most notably, the breast imaging e-learning modules available on the HHC have been used as supporting material for IAEA-organised regional breast imaging training courses, functioning as preparatory and reinforcing components.

### 3.2. Translating Evidence into Appropriate Use: The PET/CT Module as a Worked Example

**Figure 1:**
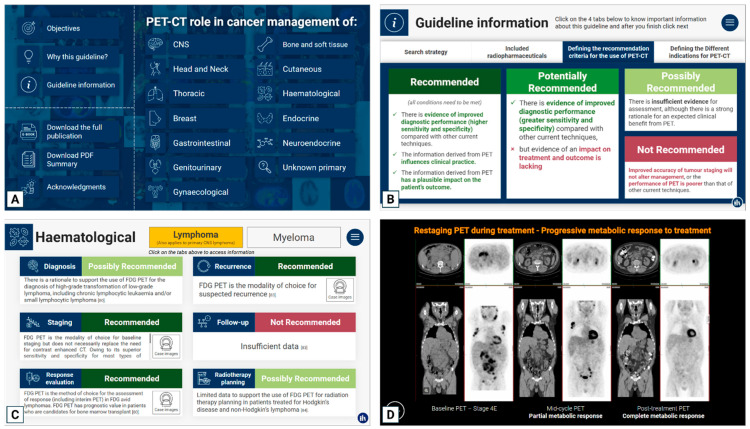
Selected screens from the IAEA e-learning module PET-CT for the Management of Cancer Patients: A Review of the Existing Evidence, illustrating its structure as a clinical decision-support tool. (**A**) Module navigation page showing coverage across tumour categories. (**B**) Recommendation framework defining when PET/CT is recommended, potentially recommended, possibly recommended, or not recommended. (**C**) A tumour-specific summary applying this framework across the main six clinical scenarios (Bracketed numbers visible refer to internal module references and do not correspond to the paper’s reference list). (**D**) A representative case from the integrated HHC PET/CT gallery.

The previously mentioned PET/CT e-learning module illustrates well how structured e-learning can support physicians in the daily decisions their work demands, as the competent use of PET/CT is not only about reading scans but also about deciding whether the examination is of added value in a particular clinical scenario or not [26]. The module is designed to support this judgement at the point of decision: it focuses on when PET/CT is beneficial for different clinical indications across a range of tumour types, drawing on the published evidence base summarised in IAEA Human Health Series No. 45 [25] and supplementing it with representative cases from the HHC PET/CT gallery (Figure 1). Hosted directly on the HHC without log-in requirements to support quick consultation as a reference resource, the module has recorded approximately 3600 visits from 2518 active users since its release in July 2025.

This is the kind of resource designed to support a nuclear medicine physician working with a newly installed PET/CT scanner and limited local subspecialty mentorship. By working through the evidence-based indications, the module aims to help the physician judge when PET/CT is likely to change management, with potential downstream benefits for contribution to multidisciplinary discussion and for the ability to cite the underlying evidence. Any such change would likely be gradual rather than transformational, and contingent on the broader clinical and educational context in which the physician practises.

### 3.3. Learning Management System Integration

A 2013 publication outlining the early development of the HHC identified learning management system (LMS) integration as a planned future step [9], realised in 2020 through integration with the IAEA’s own LMS platform, CLP4NET (Cyber Learning Platform for Network Education and Training) [27]. Beyond enabling enrolment and progress tracking, assessment management, and certification workflows, this integration introduced systematic data collection capabilities. Through CLP4NET, the Section can collect structured information on enrolment and completion rates, learner progression, and assessment performance, as well as conduct pre- and post-course surveys. The collected information is the basis on which an impact assessment framework for the e-learning activities is being developed, moving beyond basic utilisation metrics toward more structured evaluation of educational impact.

Illustrative data from one of the Section’s flagship structured courses, the IAEA Mammography Course hosted on CLP4NET [14], indicate sustained learner uptake over consecutive reporting periods: 951 enrolments and 488 certificates issued during 2024 (completion rate of 51%), and 666 enrolments and 261 certificates during 2025 (completion rate of 39%). Completion rates in this range compare favourably with those typically observed for open-access online courses in the health professions [28,29].

A mixed deployment model has been adopted: not all structured e-learning courses are hosted only within CLP4NET. Certain modules remain accessible through the HHC, removing the need for mandatory registration and supporting rapid consultation and reference use, for example, CT (Computed tomography) anatomy modules or interactive adaptations of publications. Other courses are hosted exclusively within CLP4NET when standardised tracking and certification are required, for example the Nuclear Medicine Physicians self-assessment module [30] and several courses are available on both platforms, allowing learners to choose between open exploration via the HHC and certified completion via CLP4NET.

### 3.4. Web-Based Authoring and Modernisation of Digital Materials

Recently, the Section has deliberately adopted newer responsive e-learning modules developed using Articulate Rise authoring software rather than traditional slide-based e-learning for new content development and for the revision of selected existing modules. This shift has been driven by the increasing role of smartphones and tablets as primary devices for accessing online educational resources especially in LMICs [31,32], emphasising the need for responsive design capable of adapting seamlessly to different screen sizes, since responsiveness is a critical component of usability and user experience on mobile devices [33]. Beyond device adaptability, the transition has improved visual coherence and standardised navigation across modules, with integrated multimedia, knowledge checks, and interactive blocks supporting active engagement, while the structured, block-based architecture of the software accelerates production and supports more standardised module development.

**Figure 2:**
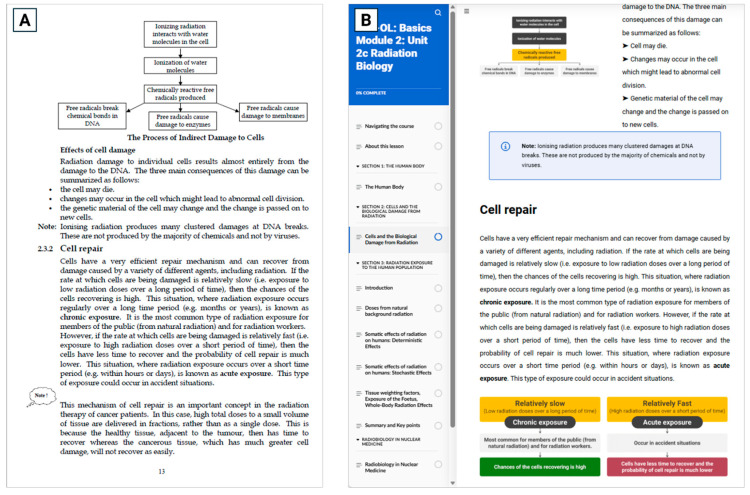
Illustration of the transformation of static reference material into interactive e-learning modules through the Section’s migration to the Articulate Rise authoring environment, using a DATOL module on radiation biology as an example. (**A**) Excerpt from the original PDF reference material, showing dense text and a static diagram. (**B**) The same content as rendered in the migrated module, incorporating a structured course navigation menu, clearer document structure, updated and new diagrams and visual blocks summarising key concepts.

Articulate Rise has also been used to modernise other digital educational resources. The PET/CT gallery—comprising 260 PET/CT cases previously distributed as static downloadable PDFs [18]—and the PDF reference material for the first three modules of the Distance Assisted Training Online for Nuclear Medicine Technologists (DATOL) programme [34] have undergone structured migration to Articulate Rise. The migration has enabled enhanced navigation, learner progress tracking, improved responsiveness, updated visuals, and the integration of multimedia and interactive elements—reflecting a broader transformation from static documents toward integrated, media-rich, interactive resources. (Figure 2) Authoring of these and other modules follows an internal e-learning framework developed by the Section, comprising authoring standards, design guidelines, and structured production workflows tailored to medical imaging education; the role of this framework in quality assurance is discussed in Section 7.

### 3.5. Case-Based Interactive Tools for Diagnostic Imaging

To enhance learner experience, the Section has developed interactive components tailored to diagnostic imaging education that can be integrated within the Articulate Rise environment. This work recognises that interactive content simulating real clinical scenarios fosters greater engagement and supports deeper knowledge acquisition [35], and that in medical imaging education this translates into case-based modules allowing learners to work through complete case scenarios in a way that reflects the clinical reading environment, bridging the gap between theoretical knowledge and real-world diagnostic practice [36,37].

**Figure 3:**
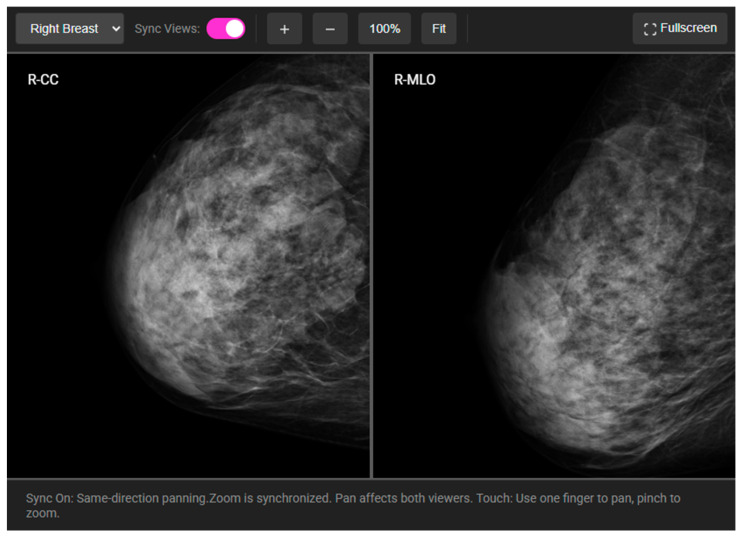
Screenshot of the mammography-specific PACS-like viewer developed for the breast imaging courses that can be embedded in Articulate Rise modules, showing synchronised right craniocaudal (R-CC) and mediolateral oblique (R-MLO) views with controls for laterality selection and view synchronization.

The first of these components is a mammography-specific PACS-like viewer developed for Parts 2 and 3 of the breast imaging courses, which comprise multimodality breast imaging cases. The viewer is built using custom HTML embedded within the Articulate Rise environment, enabling image manipulation functionality such as scrolling, panning, zooming, and toggling between views and annotations, within a user-friendly and responsive interface (Figure 3). This simulates a clinical reading workstation within a web-based module by leveraging and extending an existing capable authoring platform with imaging-specific functionality, rather than developing standalone software from scratch.

For an early-career radiologist working through this material, the educational value lies less in the volume of cases reviewed than in the structured exposure to expert reasoning about each one. Such tools are intended to support refinement of subtle finding assessment, sharpening of reporting language, and judgement about appropriate follow-up or biopsy recommendations as learners compare their own assessment against the expert interpretation provided alongside each case. This leads to expertise growth through structured exposure to variations, typical findings, borderline cases, mimics, incidental findings, artefacts, and ambiguous presentations.

Collectively, the Section’s e-learning efforts have progressed from portfolio expansion to LMS integration, platform modernization, internal quality governance, and discipline-specific innovation. Together, they reflect a coherent strategy to balance scalability, quality assurance, and clinical relevance in digital medical imaging education.

## 4. Building Networks and Connecting Experts Across Borders

For many of the questions that arise in nuclear medicine and radiology practice, including protocol decisions, ambiguous findings, and the calibration of uncertainty in a report, physicians benefit from real-time exchange with experts and peers. This is particularly true for those working in settings without local subspecialty mentorship, multidisciplinary case discussion, or established peer-review routines. Synchronous and community-based mechanisms address this gap by extending the reach of expert reasoning across borders, principally through interactive webinars and through virtual access to major international conferences via the Section’s Livestream programme. Both reach physicians through the Section’s opt-in mailing list [38], which carries webinar announcements, conference invitations and registration links, and notifications of newly released educational resources on the HHC.

### 4.1. Synchronous Learning Through Webinars

The webinar format enables real-time engagement between faculty and participants across geographically diverse Member States, allowing participants to submit questions, respond to polls, and engage in moderated discussion. Synchronous webinar-based learning is associated with improvements in knowledge acquisition and high levels of learner satisfaction when interactive elements such as live questioning and polling are incorporated. When compared to purely asynchronous formats, structured synchronous sessions enhance learner engagement and perceived learning [39]. This matters particularly in nuclear medicine and radiology, where image interpretation, protocol optimisation, and clinical decision-making involve nuanced judgement that asynchronous materials alone may not fully address. Recorded lectures convey knowledge, but case discussions and live sessions with experts demonstrate how physicians reason through clinical questions, prioritise differential diagnoses, manage uncertainty, and formulate reporting language. The international composition of faculty and participants additionally enables exchange of clinical experiences across diverse practice environments, exposing learners to heterogeneous protocols and resource settings.

Webinar topics are selected to reflect emerging needs in Member States and to align with evolving trends in nuclear medicine and radiology practice. A dedicated theranostics webinar series conducted in late 2024 and early 2025 [16] addressed practical, clinical, and implementation aspects of radionuclide therapy, with an average of 132 attendees per session representing between 41 and 65 countries.

Sessions are recorded and made available on demand within the HHC; 40 webinar recordings are currently available, extending reach and enabling asynchronous access for those unable to attend live events. This dual synchronous–asynchronous availability strengthens accessibility while preserving the educational value of live interaction.

Most recently, a flipped learning model has been adopted for selected webinars, where participants receive structured preparatory materials—such as e-learning modules—before the scheduled session. By reviewing this material in advance, more synchronous time can be devoted to application and discussion. Participants are also encouraged to submit questions during registration, enabling faculty to identify recurring themes and prepare structured responses. This shifts the emphasis from passive reception of information toward active engagement during the live session—the conditions under which expert reasoning is most visible and most useful to learners. Flipped classroom approaches, including those delivered in virtual or synchronous online environments, have been associated with increased learner participation, improved engagement, and enhanced application of knowledge when compared with traditional lecture-based formats, and have been shown to promote self-directed learning and deeper processing of complex clinical material [40,41].

The Section has collaborated with several scientific societies in the organisation of selected webinars since 2013, including the Society of Nuclear Medicine and Molecular Imaging (SNMMI), the European Association of Nuclear Medicine (EANM), the American Society of Nuclear Cardiology (ASNC), and the European Society of Radiology (ESR). The most recent of these collaborations, a joint IAEA–ESR webinar on breast imaging [42] based on the ESR’s modern radiology e-book [43], reached approximately 624 participants across online, livestream, and in-person channels, with an average attendance duration of around 57 min; in the post-webinar survey (105 respondents), the event received an overall rating of 4.68 out of 5, with 95% of respondents indicating that the format encouraged participation and 53% reporting that they expected to apply the knowledge gained in their professional practice. Qualitative feedback consistently emphasised a desire for additional case-based content, feedback consistent with the case-based orientation of the Section’s e-learning portfolio.

In this model, the IAEA contributes its global convening role and access to professionals across diverse Member States, while the scientific societies contribute specialty expertise and academic networks. By combining the reach of both institutions, these collaborations engage a broader participant base and a wider pool of expert faculty, expanding access to high-level professional dialogue across borders.

### 4.2. Conference Access Through the Livestream Programme

**Figure 4:**
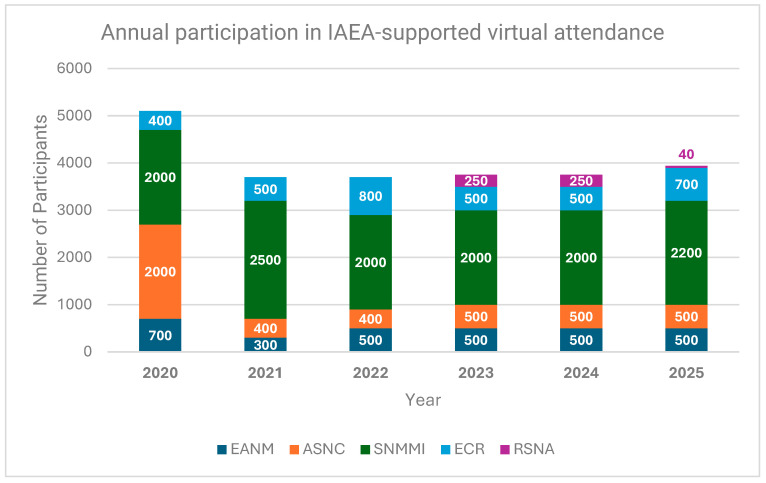
Annual number of IAEA-sponsored virtual registrations to international scientific conferences between 2020 and 2025, including the European Association of Nuclear Medicine (EANM) congress, the American Society of Nuclear Cardiology (ASNC) annual scientific session, the Society of Nuclear Medicine and Molecular Imaging (SNMMI) annual meeting, the European Congress of Radiology (ECR) and the Radiological Society of North America (RSNA) annual meeting. The elevated participation in 2020 reflects the COVID-19 pandemic context, during which scientific conferences shifted to fully virtual formats with significantly broader online accessibility than in subsequent years.

Beyond the synchronous webinars hosted on the HHC, the Section’s Livestream programme leverages the same partnerships with international scientific societies to provide medical imaging professionals from LMICs with free virtual access to major conferences organised by these societies. Between 2020 and 2025, the initiative supported virtual participation across five major international conferences, with annual participation across these events ranging between approximately 3700 and 5100 attendees per year (Figure 4).

Through sponsored virtual access, medical imaging professionals from LMICs can attend scientific sessions that might otherwise be inaccessible due to financial or logistical barriers. Participation provides CME credits, supporting professional development and maintenance of certification requirements, and enables remote engagement with current scientific discussions, emerging technologies, and expert perspectives within the global nuclear medicine and radiology community. The programme thereby helps narrow inequities in access to advanced professional education and participation in international scientific exchange across high-income and low- and middle-income settings.

### 4.3. Partnership-Based Education in the IAEA Model

Taken together, the webinar series and the Livestream programme represent the Section’s two principal mechanisms for connecting physicians to expert reasoning and to one another in real time. Both rely on partnerships with professional societies rather than on a one-way transfer of knowledge from high-income to lower-resource settings. The strongest cross-border educational models are not those in which expertise is delivered downward, but those in which physicians from different settings exchange experience, adapt content to local context, and benefit from multilingual access where possible; recognising that imaging availability, referral systems, reporting infrastructure, reimbursement, and follow-up arrangements differ across health systems and that educational content must be usable within those local realities. The Section’s reliance on co-organised activities with scientific societies, on faculty drawn from multiple regions, and on open virtual conference access reflects this orientation.

## 5. Beyond E-Learning and Webinars: Clinical Guidance and Reference Resources for Physicians

Beyond its structured e-learning modules and webinar activities, the HHC also hosts a variety of digital resources that support not only education and training but also research activities in nuclear medicine and radiology. These include IAEA publications and clinical guidance documents, teaching cases, multimedia learning resources, quality management frameworks, and databases.

IAEA publications hosted on the HHC address a broad range of topics in nuclear medicine and radiology, including clinical applications, hybrid imaging, operational practice, quality management, and global analyses of practice and capacity across different regions. These include four atlases comprising 442 hybrid imaging cases in cardiac, skeletal, and oncologic applications [19,20,21,22], as well as clinical guidance documents directly relevant to practising physicians such as the Nuclear Medicine Resources Manual [44] and the joint EANM/SNMMI/IAEA enabling guide on theranostics centre setup [23]. The HHC also aggregates links to publications authored by Section staff, organised by specialty area.

Other resources include teaching cases that illustrate imaging findings and linking theoretical knowledge to clinical practice; multimedia materials such as a 3D animated video on radionuclide therapy of neuroendocrine tumours [45] supporting both clinician and patient education; and IAEA quality management framework resources for training nuclear medicine staff in implementing quality practices.

A further example is the DATOL programme [34]. Although developed primarily as a blended-learning programme for nuclear medicine technologists, its asynchronous educational materials—including textbooks, workbooks, and exercises—are available openly through the HHC, enabling students, educators, and training institutions to incorporate them into their own teaching activities beyond the formal programme.

Together, these resources position the HHC as an open-access digital repository whose materials can be reused across geographically and institutionally diverse educational settings.

## 6. Digital Education Scope and Boundaries

Digital education in the form of online courses, webinars, case libraries, and reference materials extends the reach of expert knowledge and create opportunities for structured learning across borders, yet these cannot be a substitute for supervised clinical training, mentorship, fellowship experience, or the institutional quality routines through which physicians develop and maintain competence. The educational evidence base supports this calibrated view: systematic reviews of cognitive interventions intended to improve clinical reasoning have shown heterogeneous effects, with stronger results in simulation and knowledge-based assessments than in transferred clinical practice [46,47] and broader work on diagnostic safety has emphasised that physician decision-making is shaped by both cognitive and system factors, with educational interventions one of several elements—alongside peer review, clinical decision support, and structured results communication—that may contribute to better practice [48]. Honest framing of what digital education can and cannot achieve is therefore essential, both to set appropriate expectations for learners and to ensure that digital initiatives are designed and used as complements to clinical training rather than as alternatives to it.

Within these bounds, digital education can plausibly contribute to specific aspects of physician reasoning. In nuclear medicine, structured case-based learning can help physicians recognise inflammatory and treatment-related uptake patterns that may mimic malignancy on PET/CT. In radiology, similar formats can address satisfaction-of-search and missed subtle findings as well as the importance of comparison with prior imaging. The IAEA’s case-based modules—including the PET/CT gallery, the mammography courses, and the teaching cases and atlases hosted on the HHC—are deliberately oriented toward these kinds of targets. Table 2 summarises the kinds of claims about digital education that can be reasonably defended on the basis of available evidence and conceptual grounds, alongside the kinds of claims that go beyond current evidence.

## 7. Quality Assurance for Digital Education: The Section’s E-Learning Framework

Recognising both the value and the limits of digital education described above, the Section’s approach centres on ensuring that its courses are well designed, well governed, and capable of supporting the kinds of physician learning that digital education can plausibly contribute to.

Central to this approach is the Section’s recently developed internal e-learning framework, which comprises authoring standards, design guidelines, and structured production workflows. It addresses the elements that distinguish high-quality e-learning from generic digital content: clear learning objectives, structured navigation, consistent visual design and integration of multimedia and interactive activities. While drawing on evidence-informed international e-learning guidance developed within the United Nations system [49], this framework was specifically tailored to address the broader institutional requirements of the IAEA, the Section’s internal operational processes, and, most notably, the discipline-specific requirements of medical imaging education. Through direct involvement of in-house nuclear medicine physicians and radiologists, it incorporates guidance on the appropriate selection, presentation, and implementation of nuclear medicine and radiology images, as well as discipline-specific interactive learning activity templates tailored to diagnostic imaging education. The framework therefore functions both as a quality assurance mechanism for new content and as a vehicle for structured collaboration among subject matter experts, instructional designers, and the Section’s technical officers.

The framework’s instructional design draws on established educational models, especially the Analysis, Design, Development, Implementation and Evaluation (ADDIE) model [50], Bloom’s taxonomy for learning objectives [51], and case- and scenario-based learning approaches [52,53]. Operationally, each course is initiated with a course plan that defines purpose, target audience, learning objectives, structure, and intended deliverables, and that is signed off by the Section Head before development begins. Role and responsibility assignments—covering IAEA officers, subject matter experts, instructional designers, and e-learning developers—are documented at the start of each project, and development follows a defined sequence of phases supported by guidance documents and reusable templates for the authoring environments used. Content quality is supported through structured collaboration between subject matter experts and instructional designers, the use of imaging-specific guidance for image selection and presentation, and end-of-development checklists.

The framework is supported by the Section’s CLP4NET infrastructure previously described, which provides the data backbone for an impact assessment framework currently being developed to move beyond utilisation metrics toward more structured evaluation of educational impact.

The Section’s quality strategy is calibrated to what e-learning can credibly deliver: courses are designed and governed as resources that complement supervised clinical training, mentorship, and institutional quality assurance, with certificates documenting participation and completion rather than credentialing clinical competence.

## 8. Challenges and Future Directions

The continued expansion of the Section’s digital education activities is influenced by a set of interrelated operational, strategic, and global considerations. A central challenge lies in sustaining and scaling these initiatives within finite human and financial resources. In response, the Section is working to improve efficiency through the further adoption of standardised development frameworks, including structured templates and workflows, as well as through the continued exploration of emerging technologies—particularly AI-assisted approaches—to support and accelerate content development, while maintaining appropriate scientific oversight. Complementing these efforts, the utilisation of evaluation and impact data will help the Section to better prioritise activities and direct resources toward areas of highest educational value.

The Section has been engaging with the implications of artificial intelligence for education since 2021, when the IAEA convened a Technical Meeting on Artificial Intelligence for Nuclear Technology and Applications. Subsequent IAEA analysis of artificial intelligence in education (AIED) frames well-designed AIED as having the potential to improve learning effectiveness and reduce implementation and workload costs, while emphasising that current applications are best understood as non-autonomous systems intended to augment the work of educators and learners and that integration depends on collaboration between researchers, education practitioners, and technology developers [54,55]. At present, no AIED approaches have been systematically implemented within the HHC, reflecting both the early stage of integration and the position that such technologies should not yet be considered a first-line solution in health education. The state of the art in AIED continues to be monitored, and future exploration—particularly in areas such as adaptive learning, intelligent tutoring systems, and multilingual content delivery—will be addressed in upcoming expert and consultancy meetings on innovative instructional approaches, with careful attention to pedagogical value and ethical frameworks.

Another important consideration relates to the overall visibility and reach of the HHC, which could be increased through more active promotion and dissemination of educational activities, alongside strengthened collaboration with scientific societies to facilitate cross-promotion. In addition, the introduction of continuing professional education accreditation represents a potential avenue to enhance learner engagement, reflecting the importance of formal recognition in motivating participation in digital learning environments [56].

At the same time, both the clinical fields of nuclear medicine and radiology and the broader landscape of digital learning are undergoing rapid evolution. Advances in hybrid imaging, theranostics, and artificial intelligence, coupled with shifting learner expectations toward mobile-first and flexible formats, necessitate continuous adaptation. In this context, the Section is exploring the use of emerging innovative educational technologies, while continuing to expand interactive and simulation-based tools tailored to imaging practice. There is also a growing emphasis on case-based and modular educational formats, which allow for more rapid updating of content and align with microlearning approaches that support incremental, practice-oriented learning. A natural progression of this work is toward more deliberately blended-learning models that connect digital education with supervised clinical experience: online modules that prepare physicians ahead of regional workshops and digital follow-up resources that sustain learning after face-to-face training through refresher cases and expert discussion.

Finally, global equity considerations remain central to the design and dissemination of digital education initiatives. Variability in language, digital infrastructure, and access to reliable internet connectivity can influence how resources are utilised across Member States. To address this, efforts are directed toward expanding multilingual content, including the use of technology-assisted translation to improve scalability, as well as ensuring that educational materials are accessible across a range of devices and bandwidth conditions. Together, these approaches reflect an ongoing effort to balance scalability with inclusivity, while adapting to the diverse contexts in which digital education is applied.

## 9. Conclusions

Digital education in nuclear medicine and radiology is not the relocation of lectures to an online platform. As described in this paper, the IAEA Nuclear Medicine and Diagnostic Imaging Section’s digital education portfolio represents a coordinated strategy to extend access to specialised knowledge and to support the practising nuclear medicine physician and radiologist in the daily decisions their work demands.

Several themes emerge from this analysis. The combination of technological infrastructure with institutional governance—through the internal e-learning framework, the CLP4NET platform, and the Section’s quality assurance approach—supports the consistent development and delivery of educational content. The integration of asynchronous and synchronous formats, enhanced by partnerships with international scientific societies, extends the reach of expert reasoning beyond what either format could achieve alone. The commitment to free and open access decreases barriers to participation, particularly for physicians in low- and middle-income settings. And the progressive development of data-enabled evaluation creates the conditions under which the impact of these activities can be more rigorously assessed in future.

These efforts are not, however, a substitute for the supervised clinical training, mentorship, fellowship experience, and institutional quality routines through which physicians develop and maintain competence; they are best understood as resources that complement these other elements of physician development. Several limitations of the present analysis also warrant explicit acknowledgement. The paper is descriptive and does not constitute a formal evaluation of educational outcomes or clinical impact. While selected engagement, participation, and post-event satisfaction data are reported where available, these do not capture knowledge acquisition, retention, or translation into clinical practice at the level the literature would consider outcome data; this is a recognised gap, and a consultancy meeting on e-learning impact assessment convened by the Section in October 2025 represents an initial step toward more structured evaluation methodologies for future work. The manuscript is also authored predominantly by individuals directly engaged in the development and management of the educational initiatives described; independent external evaluation of the ecosystem remains an important direction for future work.

As digital education continues to evolve, the Section’s digital education ecosystem will continue to develop in alignment with technological advances, emerging educational models, and the evolving requirements of capacity building in the global medical imaging community.

## 10. Key Messages

- The IAEA Nuclear Medicine and Diagnostic Imaging Section’s digital education portfolio is a coordinated strategy to support diagnostic quality at the level of the practising physician, not content delivery alone.
- Open access on the Human Health Campus underpins equitable reach across 159 countries, with the majority of visits originating from low- and middle-income settings.
- Structured e-learning is the central pillar of the portfolio, allowing physicians to engage with curriculum-oriented content at their own pace and in response to immediate clinical needs.
- Webinars and Livestream conference access connect physicians who would otherwise work in professional isolation to expert reasoning and to international scientific exchange.
- Quality assurance is centred on an internal e-learning framework that combines course development standards with discipline-specific guidance from in-house nuclear medicine physicians and radiologists.
- Defensible claims about digital education must be distinguished from overreaching claims that it alone can reduce diagnostic errors, replace supervised training, or substitute credentialing.
- The Section’s digital education ecosystem is a continuously evolving response to changing clinical, educational, and global equity considerations.

## Figures and Tables

**Table 1:** Digital education resources within the Nuclear Medicine and Diagnostic Imaging Section of the Human Health Campus, with their scale, primary educational purpose, and representative examples for nuclear medicine physicians and radiologists.

Format		Count on the HHC	Primary Educational Purpose	Examples
Structured e-learning courses		30	Structured, self-paced study of foundational and updated knowledge.	PET/CT appropriate use criteria [13]; Mammography courses [14,15]
Interactive webinars		40 recordings (live sessions ongoing)	Time-bound opportunities to participate in or observe expert reasoning, current guidance, and case discussion.	Theranostics webinar series [16]
Lectures (Recorded and slide based)		395	Reference access to expert teaching across specialty areas	Cardiac nuclear medicine lectures [17]
Teaching cases (Slide based and PET/CT gallery)		357	Clinically contextualised illustration of imaging findings and diagnostic reasoning	PET/CT gallery [18]
Publications	IAEA publications	105	Reference and clinical guidance documents	Four hybrid imaging atlases [19,20,21,22]; Theranostics centre setup guide [23]
	Linked publications by section staff	591		

**Table 2:** Defensible and overreaching claims about the impact of digital education in nuclear medicine and radiology. The left column states claims that can be reasonably defended based on available evidence and conceptual grounds. The right column states claims that go beyond current evidence or misrepresent the scope of digital education.

Defensible Claim	Overreaching Claim
Improve appropriate test selection and protocol choice	Directly reduces diagnostic errors at the population level
Strengthen recognition of imaging pitfalls, mimics, and artefacts	Replaces supervised clinical training, fellowship, or observership
Improve clarity, completeness, and communication of uncertainty in reports	Substitutes for credentialing, licensure, or local regulatory approval of clinical practice
Expand peer exchange and reduce professional isolation across borders	Removes the need for local clinical supervision and peer review
Support longitudinal continuing professional development when integrated with audit and mentorship	Independently improves patient outcomes without integration into broader clinical practice

## Data Availability

No new data were created or analyzed in this study. The educational resources described in this paper are publicly available through the IAEA Human Health Campus (https://www.iaea.org/resources/hhc; accessed on 26 March 2026).

## Abbreviations

The following abbreviations are used in this manuscript:

ADDIE	Analysis, Design, Development, Implementation and Evaluation
AI	Artificial Intelligence
AIED	Artificial Intelligence in Education
ASNC	American Society of Nuclear Cardiology
CLP4NET	Cyber Learning Platform for Network Education and Training
CME	Continuing Medical Education
CT	Computed Tomography
DATOL	Distance-Assisted Training Online for Nuclear Medicine Technologists
EANM	European Association of Nuclear Medicine
ECR	European Congress of Radiology
ESR	European Society of Radiology
FDG	Fluorodeoxyglucose
HHC	Human Health Campus
IAEA	International Atomic Energy Agency
LMIC	Low- and Middle-Income Country
LMS	Learning Management System
PACS	Picture Archiving and Communication System
PET/CT	Positron Emission Tomography/Computed Tomography
R-CC	Right Craniocaudal
R-MLO	Right Mediolateral Oblique
RSNA	Radiological Society of North America
SNMMI	Society of Nuclear Medicine and Molecular Imaging
WHA	World Health Assembly
